# The hairpin region of Ndc80 is important for the kinetochore recruitment of Mph1/MPS1 in fission yeast 

**DOI:** 10.1080/15384101.2016.1148842

**Published:** 2016-02-22

**Authors:** Aldona Ewa Chmielewska, Ngang Heok Tang, Takashi Toda

**Affiliations:** a The Francis Crick Institute, Lincoln's Inn Fields, London, United Kingdom; b Hiroshima Research Center for Healthy Aging (HiHA), Department of Molecular Biotechnology, Graduate School of Advanced Sciences of Matter, Hiroshima University, Higashi-Hiroshima, Japan

**Keywords:** fission yeast, kinetochore, Mph1/MPS1, Ndc80, spindle assembly checkpoint

## Abstract

The establishment of proper kinetochore-microtubule attachments facilitates faithful chromosome segregation. Incorrect attachments activate the spindle assembly checkpoint (SAC), which blocks anaphase onset via recruitment of a cohort of SAC components (Mph1/MPS1, Mad1, Mad2, Mad3/BubR1, Bub1 and Bub3) to kinetochores. KNL1, a component of the outer kinetochore KMN network (KNL1/Mis12 complex/Ndc80 complex), acts as a platform for Bub1 and Bub3 localization upon its phosphorylation by Mph1/MPS1. The Ndc80 protein, a major microtubule-binding site, is critical for MPS1 localization to the kinetochores in mammalian cells. Here we characterized the newly isolated mutant *ndc80-AK01* in fission yeast, which contains a single point mutation within the hairpin region. This hairpin connects the preceding calponin-homology domain with the coiled-coil region. *ndc80-AK01* was hypersensitive to microtubule depolymerizing reagents with no apparent growth defects without drugs. Subsequent analyses indicated that *ndc80-AK01* is defective in SAC signaling, as mutant cells proceeded into lethal cell division in the absence of microtubules. Under mitotic arrest conditions, all SAC components (Ark1/Aurora B, Mph1, Bub1, Bub3, Mad3, Mad2 and Mad1) did not localize to the kinetochore. Further genetic analyses indicated that the Ndc80 hairpin region might act as a platform for the kinetochore recruitment of Mph1, which is one of the most upstream SAC components in the hierarchy. Intriguingly, artificial tethering of Mph1 to the kinetochore fully restored checkpoint signaling in *ndc80-AK01* cells, further substantiating the notion that Ndc80 is a kinetochore platform for Mph1. The hairpin region of Ndc80, therefore, plays a critical role in kinetochore recruitment of Mph1.

## Introduction

Proper kinetochore-microtubule attachment lies at the heart of faithful segregation of chromosomes during mitosis. The outer kinetochore KMN network (KNL1, Mis12 complex and Ndc80 complex) plays a central role in ensuring proper microtubule attachment.^1-4^ Numerous studies have shown that the Ndc80 complex binds to microtubules in a tripartite manner – through the unstructured N-tail, the CH (calponin homology) domain and the internal loop region of the Ndc80 protein.^5-11^ While the N-tail and the CH domain are responsible for lateral attachment of microtubules by directly interacting with microtubule lattice,^12-14^ the loop, which interrupts the medial coiled-coil domain, helps establish end-on attachment by indirectly interacting with the microtubule plus end through recruiting microtubule-associated proteins.^11,15,16^ Incorrectly attached or unattached kinetochores are recognized by the spindle assembly checkpoint (SAC). Upon SAC activation, the ensemble of the SAC components (Mph1/MPS1, Mad1, Mad2, Mad3/BubR1, Bub1 and Bub3) is recruited to the kinetochores, generating a ‘wait-anaphase’ signal to halt mitotic progression.^17,18^

Intriguingly, aside from its microtubule-binding activity, the Ndc80 complex has been implicated to play a role in SAC activation.^2,19-22^ In humans, MPS1 binds to the CH domain of Ndc80 through its N-terminal extension (NTE) and tetratricopeptide repeat (TPR) *in vitro*,^23^ competing for the microtubule binding sites.^24,25^ This interaction appears to be regulated by Aurora B phosphorylation of the Ndc80 and MPS1 proteins.^23,26,27^ In addition, the CH domain of Nuf2 binds to the middle region (MR) of MPS1 *in vitro*.^24,26^ However, it remains unclear as to whether the CH domains are necessary and sufficient for MPS1 recruitment to unattached kinetochores *in vivo*. It is also not known how MPS1 is recruited to the kinetochore in simple model organisms such as yeasts. In this study, through analyses of a newly isolated *ndc80* mutant (*ndc80-AK01*) in fission yeast, we show that the hairpin region, situated between the CH domain and the coiled-coil domain, plays a critical role in SAC signaling through recruitment of Mph1 to the unattached kinetochore.

## Results

### The ndc80-AK01 mutant is hypersensitive to microtubule drugs and contains a point mutation in the internal hairpin region

**Figure 1.**
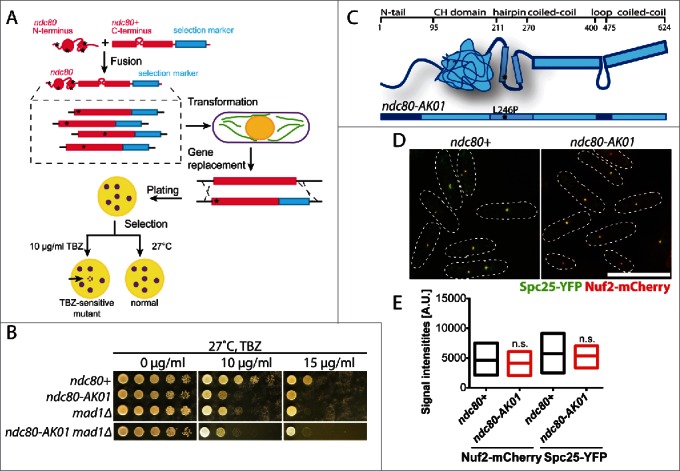
Isolation and initial characterization of the *ndc80-AK01* mutant. (A) Scheme of *ndc80* mutant isolation. Randomly mutagenized N-terminal fragments (corresponding to 1st to 280th amino acid residues) of the *ndc80* gene were fused with a C-terminal construct (238th to 624th amino acid) containing a kanamycin selection marker. The fusion *ndc80* constructs were then transformed into a wild type fission yeast strain, by which the endogenous *ndc80*^+^ gene is replaced by the mutated *ndc80-kan*^*R*^ gene through homologous recombination. Asterisks represent introduced mutations. Transformants were plated on YE5S plates at 27°C, and replica-plated onto kanamycin (G418) plates after 24 h. Upon 4 d incubation, cells were again replica-plated to YE5S with 10 μg/ml TBZ (thiabendazole). TBZ sensitive mutants cannot grow on TBZ plates. (B) TBZ sensitivity. Ten-fold serial dilutions of individual cells were spotted onto YE5S containing indicated concentrations of TBZ for 3 d at 27°C (5 × 10^4^ cells in the first spot). (C) Schematic presentation of Ndc80 protein. The *ndc80-AK01* mutant contains a mutation in the hairpin region of Ndc80 (L246P). (D) The Ndc80 complex in *ndc80-AK01* remains intact. Spc25-YFP and Nuf2-mCherry were visualized in wild type and *ndc80-AK01* after 120 minutes in YE5S with 50 μg/ml TBZ and 60 μg/ml of CBZ at 27°C. n > 200 cells. (E) Quantification of Spc25-YFP and Nuf2-mCherry signal intensities. Statistical significance was determined by student's t-test (n > 20 cells). Scale bar, 10 μm.

By isolating temperature sensitive *ndc80* mutants specifically defective in kinetochore-microtubule attachment, we previously identified the Ndc80 internal loop as an important platform for regulating microtubule attachment and timely mitotic progression.^7,9,11^ In this study, we adopted a similar screening method (Fig. 1A) to isolate mutants that are sensitive to the microtubule depolymerising drug thiabendazole (TBZ), rather than high temperature. Subsequently, we isolated the *ndc80-AK01* mutant that is TBZ-sensitive to an extent similar to *mad1*Δ (Fig. 1B). *ndc80-AK01* contains a single amino acid change (L246P) in the hairpin region that resides between the CH domain and the coiled-coil region (Figs. 1C and S1). We tested the structural integrity of the Ndc80 complex in the *ndc80-AK01* mutant by visualizing other components of the complex. As in wild type, Nuf2 and Spc25 co-localize as discrete dots in the *ndc80-AK01* mutant (Fig. 1D and E), indicating that the defective phenotypes of the *ndc80-AK01* mutant are not a result of disrupting overall architecture of the Ndc80 complex.

### The ndc80-AK01 mutant shows defects in SAC activation

**Figure 2.**
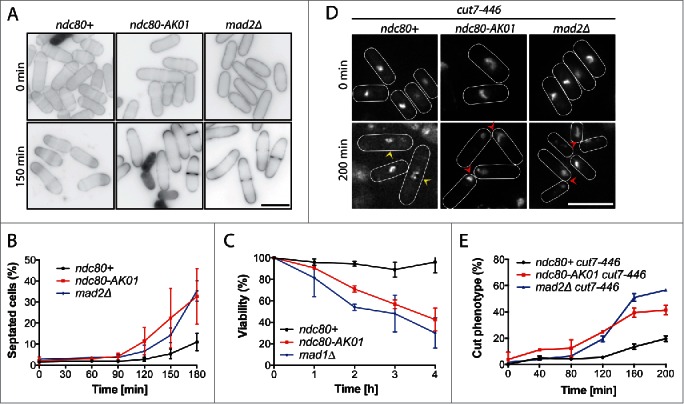
SAC signaling is defective in the *ndc80-AK01* mutant. A. Exponentially growing cells were synchronized with 12.5 mM hydroxyurea (HU), washed out and placed in YE5S medium in the presence of 50 μg/ml TBZ and 60 μg/ml of CBZ at 27°C. Samples were stained with Calcofluor. B. Quantification of septated cells. Values are averages from 3 repeats. n > 150 cells for each time point. C. Viability test. Cells were grown in YE5S containing 50 μg/ml TBZ and 60 μg/ml of CBZ at 27°C and 200–500 cells were plated on YE5S plates. After 3 d incubation, the number of viable colonies was counted. D. The *ndc80-AK01* and *mad2*Δ mutant cells display the “cut” phenotype in combination with the temperature-sensitive kinesin-5 mutant. Exponentially growing cells at 25°C were shifted up to 36°C. DAPI was used to stain DNA. Over-condensed chromosomes (yellow arrowheads) and the “cut” phenotype (red arrowheads) are marked. E. Quantification of cells showing the “cut” phenotype. Cells displaying “cut” phenotype as shown in D were quantified every 40 minutes for 200 minutes. Values are averages from 3 repeats. n > 150 cells for each time point. Error bars in B, C and E represent standard deviations. Scale bars, 10 μm.

Next, we examined the phenotypic responses of *ndc80-AK01* in the presence of microtubule drugs. We found that upon addition of TBZ and CBZ (carbendazim),^28^ *ndc80-AK01* cells displayed an increased septation index and reduced viability, compared to wild type cells (Fig. 2A–C). These responses were very similar, if not identical, to those of *mad2*Δ.

We also examined the phenotypes of *ndc80-AK01* under mitotic arrest conditions. For this purpose, we constructed double mutants between *ndc80-AK01* and the temperature sensitive *cut7-446* mutant (kinesin-5),^29^ or the *nda3-1828* mutant (β-tubulin).^30^ As reported previously, ^29^ *cut7-446* mutants displayed over-condensed chromosomes after incubating at 36°C for 200 minutes (Fig. 2D). In sharp contrast, *cut7-446 ndc80-AK01* cells, like *cut7-446 mad2*Δ cells, rapidly exited mitosis and exhibited the characteristic “cut” cell phenotype - lethal cell division in the absence of chromosome segregation (Fig. 2D and E). Likewise, we observed a reduced percentage of cells showing over-condensed chromosomes in *nda3-1828 ndc80-AK01* cells, as in *nda3-1828 mad2*Δ cells (Fig. S2A and B), suggesting that *ndc80-AK01* is defective in SAC activation. Consistent with this proposition, double mutants of *ndc80-AK01* and deletions of SAC components exhibited no additive adverse effects on growth properties and hypersensitivity to TBZ (Figs. 1B and S2C). Collectively, these results consistently indicate that the *ndc80-AK01* mutant is specifically defective in SAC signaling.

### The ndc80-AK01 mutant fails to recruit all SAC components to the unattached kinetochore

**Figure 3.**
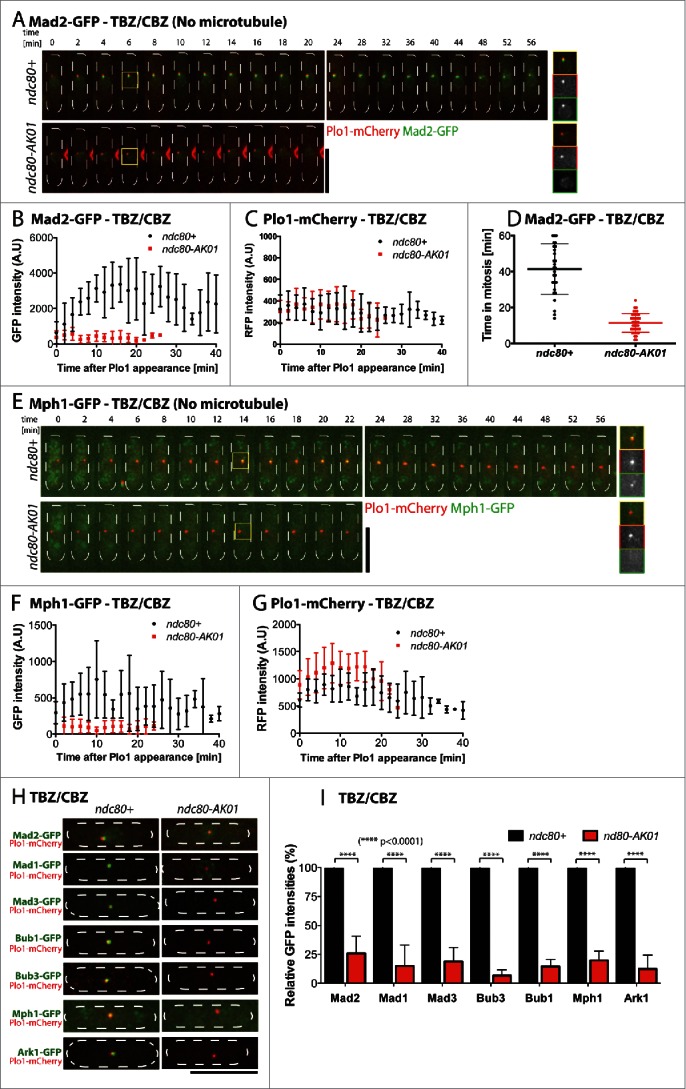
The *ndc80-AK01* mutant fails to recruit SAC components to the kinetochore under mitotic arrest condition. A.-H. Exponentially growing cells (4 × 10^6^ cells/ml) were cultured in YE5S with 50 μg/ml TBZ and 60 μg/ml of CBZ. After 30 min, live samples were placed on lectin-coated dishes and imaged for further 60 min. Imaging started after 30 minutes. Representative images of *ndc80*^+^ and *ndc80-AK01* with Mad2-GFP and Plo1-mCherry (A), Mph1 and Plo1-mCherry (E) and other SAC components (H) are shown. Quantification of signal intensities derived from Mad2-GFP (B), Mph1-GFP (F), Plo1-mCherry (C and G) and other GFP-tagged SAC components (I) are also indicated. The duration in mitosis (judged by localization of Plo1-mCherry at SPBs) was measured and quantified in D. n > 10 cells for B, C, F, and G and n > 30 cells for D. Scale bars, 10 µm.

To determine the underlying reason for the checkpoint defects seen in the *ndc80-AK01* mutant, we next observed the localization of individual GFP-tagged SAC components in the presence of TBZ/CBZ (absence of microtubules), using Plo1-mCherry as a mitotic marker (Polo kinase).^31^ Mad2 is known to be the very last component in the SAC signaling pathway that is recruited to unattached kinetochores.^18^ In the presence of TBZ/CBZ, wild type cells displayed a long mitotic delay with prolonged Mad2-GFP localization to the kinetochores (Fig. 3A–D). In contrast, we did not observe analogous Mad2-GFP kinetochore signals or mitotic delay in the *ndc80-AK01* mutant (Fig. 3A–D).

Mph1 kinase, on the other hand, is one of the most upstream SAC components.^31^ As in the case of Mad2-GFP, recruitment of Mph1-GFP to the kinetochores was also impaired in the *ndc80-AK01* mutant (Fig. 3E–G). Consistent with this notion, systematic analyses of the other SAC components, including Bub1, Bub3, Mad3 and Mad1, revealed that these proteins are also mis-localized from the kinetochores in *ndc80-AK01* under mitotic arrest conditions (Fig. 3H, I and S3A–H).

In fission yeast, as in human cells, Ark1/Aurora B is assigned as the most upstream component.^31^ However, its kinetochore/centromere localization substantially depends on Bub1 through positive feedback regulation; Bub1 phosphorylates histone H2A, thereby recruiting Sgo2/Shugoshin that in turn promotes Ark1/Aurora B localization.^28,32,33^ In line with this model, we found reduced localization of Ark1-GFP to the kinetochores/centromeres in *ndc80-AK01*, to a similar extent as in *bub1*Δ cells (Fig. S4). Together, we concluded that the primary defects observed in the *ndc80-AK01* mutant can be attributed to impaired Mph1 recruitment to kinetochores, which leads to failure in recruitment of the other SAC components and abortive mitotic arrest.

### Artificial tethering of Mph1 to the kinetochore arrests ndc80-AK01 cells in mitosis

**Figure 4.**
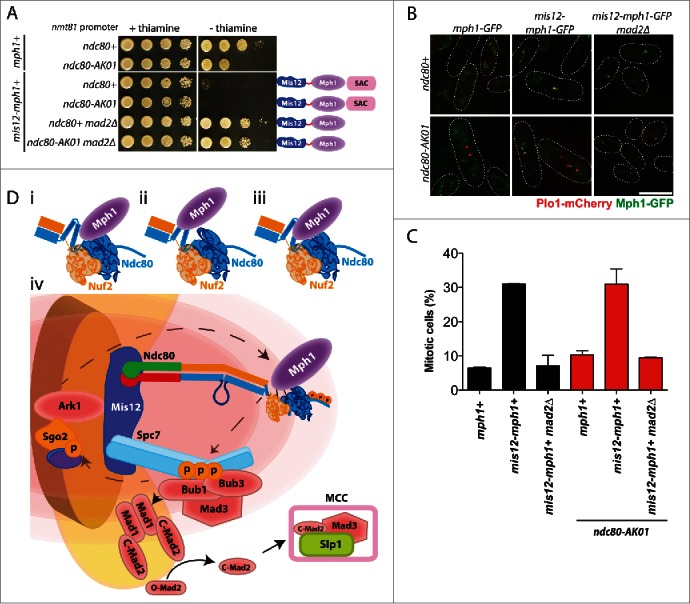
Tethering of Mph1 to the kinetochore rescues the SAC signaling defects in the *ndc80-AK01* mutant. A. Rescue of the SAC signaling defects in *ndc80-AK01* by kinetochore-tethered Mis12-Mph1. Spot tests of the indicated strains were performed as in Fig. 1B. B. Confirmation of Mis12-Mph1-GFP localization to the kinetochores. Representative images of cells grown in the absence of thiamine are shown. Mitotic cells were identified by the presence of Plo1-mCherry at SPBs. C. Quantification of mitotic cells in the indicated strains. The percentage of mitotic cells was counted using the presence of Plo1-mCherry at SPBs as a mitotic marker. n > 200 cells. D. Mph1 is recruited to the Ndc80 complex at unattached kinetochores. Mph1 potentially localizes to the CH domain (i), the hairpin region (ii) or both (iii). (iv) Scheme of SAC components recruitment to unattached kinetochores in fission yeast. Ark1/Aurora B is recruited to the centromere/kinetochore (in the figure, Bub1-mediated phosphorylated histone H2A is denoted by purple oval adjacent to Sgo2) and Mph1 localizes to the Ndc80 complex. Once at the kinetochore, Mph1 phosphorylates Spc7 leading to the recruitment of Bub1, Bub3 and Mad3, which is critical to recruit the Mad1-Mad2 heterotetramer complex to the kinetochore. The sequential conformation changes of Mad2 result in the formation of the MCC (C-Mad2-Mad3-Slp1/Cdc20) and SAC activation. Scale bar, 10 μm.

If compromised Mph1 recruitment to unattached kinetochores were the main defect in *ndc80-AK01*, it would lead to a critical prediction that artificial tethering of Mph1 to the kinetochore should restore SAC signaling. In fission yeast, as in other species,^23,34^ Mph1 tethering to the kinetochore in wild type cells, but not in *mad2*Δ, results in constitutive checkpoint activation with a high mitotic index.^28,31,35^ Accordingly, we constructed strains that contained Mph1 fused to Mis12 under the thiamine-repressible *nmt81* promoter (provided by Silke Hauf) in the *ndc80-AK01* background. Intriguingly, Mis12-Mph1 cells, irrespective of *ndc80*^+^ or *ndc80-AK01*, showed robust growth inhibition, which depended upon the lack of thiamine (Fig. 4A). Observation of liquid cultures indicated that growth retardation was due to prolonged mitotic arrest (Fig. 4B and C). Consistent with SAC activation under this condition, harmful effects - both poor growth on solid plates and a high mitotic index - were reversed by the deletion of the SAC component Mad2 (Fig. 4A–C). These results were entirely consistent with the idea that the primary, if not sole, defect of *ndc80-AK01* is ascribable to its inability to recruit Mph1 to the unattached kinetochore. Hence, we propose that the hairpin region situated between the CH domain and the coiled-coil domain plays a crucial role in SAC signaling through recruitment of Mph1 when proper attachment of the kinetochore to spindle microtubules fails to be established.

## Discussion

Our data firmly establish that a single point mutation within the hairpin region of Ndc80 inhibits recruitment of Mph1 to the kinetochore in fission yeast. Interestingly, an earlier report in budding yeast suggested physical interaction between Ndc80 and Mps1,^36^ and more recent work in human cultured cells show that the CH domains of Ndc80 and Nuf2 directly bind MPS1/Mph1, thereby being responsible for MPS1 localization to the unattached kinetochore.^23-26^ Given these results, we envision the following 3 scenarios to account for our findings. The first possibility is that, as in humans and possibly budding yeast,^34,36^ the CH domain of Ndc80 is a direct binding site for Mph1 in fission yeast. However, the hairpin region might be important for structural integrity of the adjacent CH domain (Fig. 4Di). The *ndc80-AK01* mutant contains a substitution from leucine to proline at the 246th amino acid situated within the αH helix. This mutation may create an abnormal kink in the structure, thereby shielding the Mph1 binding site within the CH domains of Ndc80 or Nuf2 (Fig. 4Di).

The second scenario is that fission yeast has undergone an evolutionary diversification, by which the hairpin region instead of the CH domain is solely responsible for Mph1 recruitment to the kinetochore (Fig. 4Dii). We tried immunoprecipitation between Ndc80 and Mph1, but so far we could not obtain any positive data; even using mitotically arrested wild type cell lysates, we have been unable to show interaction between Ndc80 and Mph1. It is of note that in human cell lysates, physical interaction between MPS1 and Ndc80 has also not been shown.^23-26^ We surmise that binding of these 2 proteins upon SAC activation would be transient and/or unstable *in vivo*. The third possibility is that both the CH domains and the hairpin region are required for Mph1 recruitment to the kinetochore *in vivo* (Fig. 4Diii). Multiple functional motifs have been identified in the N-terminal domain of Mph1/MPS1; the NTE and MR regions of Mph1/MPS1, respectively, have been shown to interact with each CH domain within Ndc80 and Nuf2.^24^ Therefore, it is possible that the Mph1/MPS1 N-terminal domain also interacts with the Ndc80 hairpin that is required for Mph1/MPS1 recruitment. It is of note that, despite not mutually exclusive with any possibility mentioned above, the L246P replacement may render the Ndc80-AK01 protein unable to sense a lack of tension/attachment at the kinetochore.

Regardless of several reports on the requirement of the CH domains for MPS1 recruitment to the kinetochore,^23-26^ the precise roles of the adjacent hairpin region remain to be determined in any organisms, though the hairpin seems conserved across eukaryotic organisms. Interestingly, in budding yeast, the hairpin is proposed to be important for protein-protein interactions by securing overall structures of Ndc80, although the involvement of the hairpin region in SAC signaling has not been appreciated.^37^ We postulate that the hairpin region directly or indirectly interacts with the nearby CH domains, thus creating an efficient binding pocket for MPS1/Mph1 when microtubules do not interact with the Ndc80 complex.

Although a detailed mode of interaction between Mph1 and Ndc80 has not been solidified, our current study complements the idea that Ndc80 is indeed a crucial platform for Mph1 recruitment as in other organisms. We propose a model to summarize the current understanding of SAC component recruitment to the kinetochore (Fig. 4Div). Ark1/Aurora B localizes to the centromeric region, followed by Mph1/MPS1 recruitment to the Ndc80 complex.^31^ Once there, Mph1/MPS1 phosphorylates MELT motifs in Spc7/KNL1, where the Bub1-Bub3 complex, as well as Mad3/BubR1, would dock.^28,38^ The localization of these upstream components leads to recruitment of the Mad1-Mad2 complex to the kinetochore and formation of the mitotic checkpoint complex, which inhibits mitotic progression.

## Materials and methods

### Fission yeast culture and genetics

All yeast strains used in the study are listed in Table S1. Cells were grown and maintained in standard conditions using rich YE5S medium.^39^ The experiments using *nmt* promoters were carried out in Edinburgh Minimal Media (EMM) supplemented with the required amino acids and 2 mM thiamine for cell growth. Upon washout of the reagent using a filtration system, the cells were grown in the absence of thiamine for 18-24 h.

Cells were grown at 27°C, unless otherwise stated. Spot tests were performed after adjusting cell concentration to 2 × 10^7^ cells/ml. Subsequent 10-fold dilutions were spotted on the appropriate plates containing rich YE5S medium in the presence or absence of various concentrations of TBZ or EMM with supplements or with or without 2 mM thiamine.

### Yeast strain construction

Genomic DNA from the *ndc80*^+^*-kan*^*R*^ strain^7,11^ was extracted as described previously.^7,11^ The N-terminal fragment (corresponding to 1^st^ to 280^th^ amino acid residues) of *ndc80*^+^*-kan*^*R*^ was randomly mutagenized using “error prone PCR” with unbalanced dNTP (10x dGTP excess compared to other dNTPs) using Vent DNA polymerase (New England Biolabs). The C-terminus (amino acid 238 to 624) of *ndc80*^+^*-kan*^*R*^ was amplified by PCR using PrimeSTAR polymerase (TaKaRa). The N- and C- terminal fragments were gel purified and fused together in an equimolar ratio using PrimeSTAR polymerase. Then the amplified fusion fragments were concentrated by ethanol precipitation and transformed into a wild type strain. Kanamycin (G418)-resistant clones were selected and then replica-plated onto YE5S containing 10 μg/ml thiabendazole (TBZ). We screened approximately 10,000 colonies and isolated 5 mutants judged as TBZ-sensitive. These isolates were backcrossed to check for cosegregation of the drug marker with TBZ sensitivity. Nucleotide sequencing was performed to confirm the position of the mutation within the *ndc80* gene.

### Mitotic arrest conditions

To accumulate cells in mitosis, cultures were synchronized in S phase using 12.5 mM of hydroxyurea (HU) at 25°C for 4 h. Cultures were filtered and released into HU-free YE5S media with 50 μg/ml TBZ and 60 μg/ml CBZ.^28^ Alternatively, strains were crossed with a *cut7-446* conditional mutant^29^ or an *nda3-1828* strain.^30^ Cells were grown at 25°C overnight until cultures reached mid-log phase and then shifted to 36°C. Cells were observed either upon fixation or by live imaging.

### Fluorescence microscopy

Samples were fixed with 1.6% paraformadehyde at 27°C, unless otherwise specified. Live cells were imaged on lectin-coated, glass dishes (MatTek, Ashland, MA). Images were acquired using an Olympus IX70 PlanoApo 100x, NA 1.4, oil immersion objective on an Olympus IX70 wide-field inverted epifluorescence microscope. The DeltaVision-softWoRx system (softWoRx 3.3.0; Applied Precision Co.) with a Coolsnap HQ (Roper Scientific) camera was used for acquisition of all fluorescence microscopy data. Images were taken at 14 positions along the z-axis at 0.3 μm intervals. Images obtained were deconvolved, compressed into a projection using the DeltaVision (DeltaVision-SoftWoRx; Applied Precision Ltd) maximum intensity algorithm. The maximum signal intensities, after subtracting background signals in close proximity to the fluorescent spot, were quantified. Subsequent image processing was performed in Adobe Photoshop CS5 and Adobe Illustrator CS5.1.

## Statistical data analysis

All data represent the mean of multiple experiments +/− SD. Experiment sample numbers and the number of replicates used for statistical testing have been described in the corresponding figure legends. All p-values were calculated using 2-tailed unpaired student t-tests. We followed this key for asterisk placeholders for p-values in the figures: ****p < 0.0001.

## Supplementary Material

1148842_Supplemental_Material.pdf

## Abbreviations

*SAC*: spindle assembly checkpoint
*TBZ*: thiabendazole
*CBZ*: carbendazim
*HU*: hydroxyurea
*CH*: calponin homology
