# Supplementary material for: The hairpin region of Ndc80 is important for the kinetochore recruitment of Mph1/MPS1 in fission yeast
Source: Cell Cycle. 2016 Feb 22;15(5):740–7. doi: 10.1080/15384101.2016.1148842 (PMC4845937; doi:10.1080/15384101.2016.1148842)
Supplement: 1148842_Supplemental_Material.pdf [file kccy-15-05-1148842-s001.pdf]

Supplementary Material

The hairpin region of Ndc80 is important for the kinetochore recruitment of Mph1/MPS1 in fission yeast

Aldona Ewa Chmielewska , Ngang Heok Tang and Takashi Toda

Supplementary Table S1

Supplementary Figure legends

Figure S1. *ndc80-AK01* contains a single point mutation in the internal hairpin region.

Figure S2. *ndc80-AK01* is defective in the SAC

Figure S3. Mad1, Mad3, Bub1 and Bub3 are mis-localized from the kinetochore in *ndc80-AK01*

Figure S4. Ark1-GFP does not localize to the unattached kinetochores in both *ndc80-AK01* and *bub1Δ* cells.

Supplementary Figures 1-4

Supplementary Table S1. List of strains used in the study.

Strain	*Genotype*	Figures used
AEK513	*h*$^-$ *ndc80-AK01-kan*^{R} *mad1::ura4*$^+$ *leu1 ura4*	1B
513	*h*$^-$ *leu1 ura4*	1B, 2A-C
AEK423	*h*$^-$ *mad1::ura4*$^+$ *leu1 ura4*	1B, 2C
AEK004	*h*$^-$ *ndc80-AK01-kan*^{R} *leu1 ura4 his2*	1B, 2A-C
AEK220	*h*$^+$ *spc25*$^+$*-YFP-nat*^{R} *nuf2*$^+$*-mCherry-ura4*$^+$ *cut12*$^+$*-2CFP-hph*^{R} *leu1 ura4 his2*	1D-E
AEK342	*h*$^-$ *spc25*$^+$*-YFP-nat*^{R} *nuf2*$^+$*-mCherry-ura4*$^+$ *cut12*$^+$*-2CFP-hph*^{R} *ndc80-AK01-kan*^{R}	1D-E
AEK053	*h*$^-$ *mad2::LEU2 leu1 ura4*	2A-B
AEK156	*h*$^+$ *ndc80-AK01-kan*^{R} *cut7-446 leu1 his2*	2D-E
AEK051	*h*$^+$ *cut7-446 leu1 his2*	2D-E
AEK150	*h*$^+$ *cut7-446 mad2::LEU2 his2*	2D-E
AEK595	*h*$^-$ *ndc80-AK01-kan*^{R} *mad2*$^+$*-GFP-LEU2 plo1*$^+$*-mCherry-hph*^{R} *ura4*	3A-D, 3H-I
AEK505	*h*$^-$ *plo1*$^+$*-mCherry-hph*^{R} *mad2*$^+$*-GFP-LEU2 leu1 lys1*	3A-D,3H-I
AEK531	*h*$^-$ *mph1*$^+$*-S(GGGGS)3-GFP<<kan*^{R} *plo1*$^+$*-mCherry-hph*^{R} *leu1*	3E-I
AEK709	*h*$^+$ *ndc80-AK01-kan*^{R} *mph1*$^+$*-S(GGGGS)3-GFP<<kan*^{R} *plo1*$^+$*-mCherry-hph*^{R} *leu1 ura4 his2*	3E-I
AEK700	*h*$^-$ *leu1 ark1*$^+$*-GFP<<kan*^{R} *plo1*$^+$*-mCherry-hph*^{R} *ndc80-AK01-kan*^{R}	3H-I, S4
AEK704	*h*$^-$ *plo1*$^+$*-mCherry-hph*^{R} *ark1*$^+$*-GFP<<kan*^{R} *leu1 lys1*	3H-I, S4
AEK501	*h*$^-$ *plo1*$^+$*-mCherry-hph*^{R} *mad1*$^+$*-GFP<<kan*^{R} *lys1 ura4*	3H-I, S3A-B
AEK618	*h*$^-$ *ndc80-AK01-kan*^{R} *plo1*$^+$*-mCherry-hph*^{R} *mad1*$^+$*-GFP<<kan*^{R} *ura4 ade6-210 lys1*	3H-I, S3A-B
AEK526	*h*$^+$ *plo1*$^+$*-mCherry-hph*^{R} *mad3*$^+$*-GFP<<kan*^{R} *leu1 his2*	3H-I, S3C-D
AEK641	*h*$^-$ *mad3*$^+$*-GFP<<kan*^{R} *ndc80-AK01-kan*^{R} *plo1*$^+$*-mCherry-hph*^{R} *leu1*	3H-I, S3C-D
AEK590	*h*$^+$ *ndc80-AK01-kan*^{R} *bub1*$^+$*-GFP-kan*^{R} *plo1*$^+$*-mCherry-hph*^{R} *his2 ade6-210 ura4 lys1*	3H-I, S3E-F
AEK600	*h*$^+$ *bub1*$^+$*-GFP-kan*^{R} *plo1*$^+$*-mCherry-hph*^{R} *his2 ade6-210 leu1 lys1 ura4*	3H-I, S3E-F
AEK555	*h*$^-$ *bub3*$^+$*-S(GGGGS)3-GFP<<kan*^{R} *plo1*$^+$*-mCherry-hph*^{R} *leu1*	3H-I, S3G-H
AEK634	*h+ bub3*$^+$*-S(GGGGS)3-GFP<<kan*^{R} *ndc80-AK01-kan*^{R} *plo1*$^+$*-mCherry-hph*^{R} *leu1 his2*	3H-I, S3G-H
AEK1031	*h*$^+$ *hph*^{R}*<<Pnm81<<mis12*$^+$*-(GGSG)2-mph1*$^+$*-S(GGGGS)3-GFP<<kan*^{R} *plo1*$^+$*-mCherry<<nat*^{R} *ndc80-AK01-kan*^{R} *leu1 ade6-M216 ura4 his2*	4A-C
AEK1036	*h*$^-$ *hph*^{R}*>>Pnmt81>>mph1*$^+$*-S(GGGGS)3-GFP<<kan*^{R} *plo1*$^+$*-mCherry<<nat*^{R} *ndc80-AK01-kan*^{R} *leu1*	4A-C
AEK1107	*h*$^-$ *hph*^{R}*<<Pnmt81<<mis12*$^+$*-(GGSG)2-mph1*$^+$*-GFP<<kan*^{R} *mad2Δ::hph*^{R} *plo1*$^+$*-mCherry-nat*^{R} *ndc80-AK01-kan*^{R} *leu1*	4A-C
AEK986	*h*$^-$ *hph*^{R}*>>Pnmt81>>mph1*$^+$*-S(GGGGS)3-GFP<<kan*^{R} *plo1*$^+$*-mCherry<<nat*^{R} *leu1 ade6-M216?*	4A-C
AEK990	*h*$^-$ *hph*^{R}*<<Pnm81<<mis12*$^+$*-(GGSG)2-mph1*$^+$*-S(GGGGS)3-GFP<<kan*^{R} *plo1*$^+$*-mCherry<<nat*^{R} *leu1 ade6-M216*	4A-C
AEK991	*h*$^-$ *hph*^{R}*<<Pnmt81<<mis12*$^+$*-(GGSG)2-mph1*$^+$*-S(GGGGS)3-GFP<<kan*^{R} *mad2Δ::hph*^{R} *plo1*$^+$*-mCherry-nat*^{R} *leu1*	4A-C
AEK383	*h*$^+$ *nda3-1828 leu1 ura4 his2*	S2A-B
AEK400	*h*$^-$ *ndc80-AK01-kan*^{R} *nda3-1828 leu1 ura4*	S2A-B

AEK403	*h*$^{-}$ *nda3-1828 mad2::LEU2 leu1 ura4*	S2A-B
AEK424	*h*$^{+}$ *mph1::ura4*$^{+}$ *leu1 ura4 ade6-210*	S2C
AEK425	*h*$^{-}$ *bub1::LEU2 ura4 ade6-216*	S2C
AEK468	*h*$^{-}$ *bub3::ura4*$^{+}$ *leu1 ura4*	S2C
AEK507	*h*$^{-}$ *ndc80-AK01-kan*^{R} *mph1::ura4*$^{+}$ *leu1 ura4 ade6-210*	S2C
AEK510	*h*$^{-}$ *ndc80-AK01-kan*^{R} *bub1::LEU2 ura4 leu1 ade6-M216*	S2C
AEK517	*h*$^{-}$ *ndc80-AK01-kan*^{R} *mad3::ura4*$^{+}$ *leu1 ura4*	S2C
AEK521	*h*$^{-}$ *ndc80-AK01-kan*^{R} *bub3::ura4*$^{+}$ *leu1 ura4*	S2C
AEK586	*h*$^{-}$ *ndc80-AK01-kan*^{R} *mad2::LEU2 leu1 ura4 lys1*	S2C
AEK659	*h*$^{-}$ *mad3::ura4*$^{+}$ *leu1 ura4*	S2C
AEK996	*h*$^{-}$ *bub1::LEU2 plo1*$^{+}$*-mCherry-hph*^{R} *ark1*$^{+}$*-GFP-kan*^{R} *leu1 ura4*	S4

his2 = his-2-245; leu1 = leu1-32; ura4 = ura4-D18; lys1 = lys1-131

Supplementary Figure S1. *ndc80-AK01* contains a single point mutation in the internal hairpin region.

Alignment of Ndc80 amino acid sequences from 7 different species: *M. musculus*, *R. norvegicus*, *H. sapiens*, *G. gallus*, *X. laevis*, *S. pombe* and *S. cerevisiae*. Data was obtained from UniProt database and aligned using ClustalX online. Only the region surrounding the ndc80-AK01 mutant allele is presented.

Supplementary Figure S2. *ndc80-AK01* is defective in the SAC.

A. Exponentially growing cells were shifted from 27˚C to 36˚C and samples were taken every 40 minutes for 240 minutes. Cells were then stained with DAPI and chromosome over-condensation (yellow arrowhead) was quantified. Scale bar, 10 μm.

B. Quantification of cells displaying over-condensed chromosomes. For each time point, more than 150 cells were counted. The values are averages from two experiments.

C. A serial dilution assay of strains used in the current study. The number of initial spot cells (far-left) was 5 x 10^4 cells. Cells were plated on YE5S, YE5S containing 10 and 15 μg/ml TBZ at 27˚C for 3 d.

Supplementary Figure S3. Mad1, Mad3, Bub1 and Bub3 are mis-localized from the kinetochore in *ndc80-AK01*.

A-H. Exponentially growing cells were placed in YE5S containing 50 μg/ml TBZ and 60 μg/ml of CBZ at 27˚C. After 30 min, aliquots of cells were placed on lectin-coated dishes and imaged for 60 min. Representative images of wild type and *ndc80-AK01* cells that contain Mad1-GFP and Plo1-mCherry (**A**), Mad3-GFP and

Plo1-mCherry (**C**), Bub1-GFP and Plo1-mCherry (**E**), Bub3-GFP and Plo1-mCherry (**G**) are shown. Scale bar, 10 μm. Quantification of GFP signal intensities of each SAC components is presented in **B** (Mad1-GFP), **D** (Mad3-GFP), **F** (Bub1-GFP) and **H** (Bub3-GFP). n > 10 cells.

Supplementary Figure S4. Ark1-GFP does not localize to the unattached kinetochores in both *ndc80-AK01* and *bub1Δ* cells.

A. Representative images of wild type, *bub1Δ* and *ndc80-AK01* cells that contain Ark1-GFP and Plo1-mCherry are shown. Scale bar, 10 μm.

B. Quantification of Ark1-GFP signal intensities. n > 10.

C. The duration of mitosis judged by Plo1-mCherry signals at the SPB. n > 30.
